# High-fat diet-disturbed gut microbiota-colonocyte interactions contribute to dysregulating peripheral tryptophan-kynurenine metabolism

**DOI:** 10.1186/s40168-023-01606-x

**Published:** 2023-07-19

**Authors:** Penghao Sun, Mengli Wang, Yong-Xin Liu, Luqi Li, Xuejun Chai, Wei Zheng, Shulin Chen, Xiaoyan Zhu, Shanting Zhao

**Affiliations:** 1 College of Veterinary Medicine, Northwest A&F University, Yangling, 712100 Shaanxi China; 2 Shenzhen Branch, Guangdong Laboratory of Lingnan Modern Agriculture, Genome Analysis Laboratory of the Ministry of Agriculture and Rural Affairs, Agricultural Genomics Institute at Shenzhen, Chinese Academy of Agricultural Sciences, Shenzhen, 518120 Guangdong China; 3 Life Science Research Core Services, Northwest A&F University, Yangling, 712100 Shaanxi China; 4 College of Basic Medicine, Xi’an Medical University, Xi’an, 710000 Shaanxi China; 5 College of Resources and Environment Sciences, Northwest A&F University, Yangling, 712100 Shaanxi China

**Keywords:** High-fat diet, Gut microbiota, Tryptophan metabolism, Kynurenine, Mitochondrial dysfunction

## Abstract

**Background:**

Aberrant tryptophan (Trp)-kynurenine (Kyn) metabolism has been implicated in the pathogenesis of human disease. In particular, populations with long-term western-style diets are characterized by an excess of Kyn in the plasma. Host-gut microbiota interactions are dominated by diet and are essential for maintaining host metabolic homeostasis. However, the role of western diet-disturbed gut microbiota-colonocyte interactions in Trp metabolism remains to be elucidated.

**Results:**

Here, 4-week-old mice were fed with a high-fat diet (HFD), representing a typical western diet, for 4 weeks, and multi-omics approaches were adopted to determine the mechanism by which HFD disrupted gut microbiota-colonocyte interplay causing serum Trp-Kyn metabolism dysfunction. Our results showed that colonocyte-microbiota interactions dominated the peripheral Kyn pathway in HFD mice. Mechanistically, persistent HFD-impaired mitochondrial bioenergetics increased colonic epithelial oxygenation and caused metabolic reprogramming in colonites to support the expansion of *Proteobacteria* in the colon lumen. Phylum *Proteobacteria*-derived lipopolysaccharide (LPS) stimulated colonic immune responses to upregulate the indoleamine 2,3-dioxygenase 1 (IDO1)-mediated Kyn pathway, leading to Trp depletion and Kyn accumulation in the circulation, which was further confirmed by transplantation of *Escherichia coli* (*E.coli*) indicator strains and colonic IDO1 depletion. Butyrate supplementation promoted mitochondrial functions in colonocytes to remodel the gut microbiota in HFD mice, consequently ameliorating serum Kyn accumulation.

**Conclusions:**

Our results highlighted that HFD disrupted the peripheral Kyn pathway in a gut microbiota-dependent manner and that the continuous homeostasis of gut bacteria-colonocytes interplay played a central role in the regulation of host peripheral Trp metabolism. Meanwhile, this study provided new insights into therapies against western diet-related metabolic disorders.

Video Abstract

**Supplementary Information:**

The online version contains supplementary material available at 10.1186/s40168-023-01606-x.

## Introduction

Tryptophan (Trp) is an essential amino acid obtained exclusively from dietary intake [1]. Trp and its metabolites exhibit critical roles in various physiological events, ranging from cell proliferation to the coordination of organismic physiological homeostasis [2]. The concentration of free Trp in the organism is governed by the activity of several Trp metabolic pathways. Approximately more than 95% of free Trp is metabolized via the kynurenine (Kyn) pathway [2, 3], and its metabolites are involved in inflammation [4], immune response [5], and excitatory neurotransmission [6]. The aberrant activation of peripheral Kyn pathway is thought to be implicated in the onset and development of several psychiatric and mental disorders (e.g., depression and schizophrenia) [6]. In addition, due to the intricate relationship between Kyn metabolites and immune responses, Kyn is gaining recognition as a mediator in several diseases such as inflammatory bowel disease, obesity, and cancer [1, 2].

The gut commensal microbiota is a critical regulator of human physiological homeostasis [7]. Among the range of regulatory processes, many are mediated by microorganism-derived metabolites or by environmental and host molecules transformed by microbes [8, 9]. Accumulating evidence indicates that Trp exerts a pivotal and unique role among a range of metabolites that constitute the bidirectional communication between gut microorganisms and the host [9]. Meanwhile, massive evidence has shown that enhanced Kyn concentration in the circulation may be attributed to the upregulated indoleamine 2,3-dioxygenase 1 (IDO1) [10, 11], a rate-limiting enzyme in the Kyn pathway that is primarily expressed in the immune system and mucosal tissues such as the gut [2]. The role of gut bacteria in controlling the activity of intestinal IDO1 has been well established in germ-free mice [9]. Additionally, some gut bacteria encode enzymes homologous to the eukaryotic Kyn pathway and are therefore capable of producing Kyn and downstream metabolites such as 3-hydroxykynurenine (3-HK) [12], which readily crosses the blood–brain barrier and exhibits neurotoxic effects [13]. However, the effect of compositional changes in the gut microbiota in response to dietary patterns on Trp metabolism has not been fully elucidated.

Diet is one of the core factors influencing human health and the etiology of many non-communicable chronic diseases [14]. Over the past few decades, dietary patterns high in fat and sucrose and low in fiber, also known as the Western-style diet, have become increasingly common around the world [15]. One definitive demonstration of the impact of changes in dietary patterns on human health is the increasing incidence of metabolic diseases in the transition from traditional non-industrial regions to Western societies [16]. Cross-regional studies of the human microbiome have shown that dietary habits significantly affect the composition and richness of the gut microbiota and that different dietary components shape gut microbial communities in a time-dependent manner [17]. An increasing number of studies have shown that almost all diet-related chronic diseases are associated with the microbiome, supporting the fact that the microbiome acts as a vector and risk factor in mediating the onset and progression of diet-related diseases [18, 19]. For example, it is known from human studies and animal models that a high-fat diet (HFD) can influence gut microbial pathogenesis to exacerbate chronic inflammation and the severity of inflammatory diseases [20]. Nonetheless, the contribution of gut bacteria in diet-induced systemic metabolic disorders, particularly amino acid metabolism, is becoming increasingly clear but remains poorly understood.

The present study aimed to investigate the mechanistic links between gut dysbiosis and disturbance of Trp-Kyn metabolism using the HFD animal model. Consistent with human epidemiological findings [21], in the present study, we found that long-term HFD disturbed serum Trp metabolism, characterized by Trp depletion and upregulation of the Kyn pathway, which correlated strongly with the expansion of phylum *Proteobacteria* in the colon. The dysregulated Kyn metabolism was abolished by antibiotic treatment in HFD-fed mice, but was transferred to standard diet-fed mice by fecal microbiota transplantation (FMT), indicating a causal role of the gut microbiota in HFD-disturbed Trp metabolism. Given the substantial role of the IDO1 in the Trp-Kyn pathway, we hypothesized that interactions between HFD-altered gut microbiota and colonocytes contribute to the HFD-induced dysregulation of Trp metabolism. Colon RNA sequencing analysis revealed that *Proteobacteria*-derived lipopolysaccharide (LPS) triggered a significant colonic inflammatory response, upregulating the expression of IDO1 in colonic tissue to promote serum Kyn concentrations, which was further confirmed by transplantation of *Escherichia coli* (*E. coli*) indicator strains and colonic IDO1 depletion. Having established the central role of gut bacteria in regulating systemic Trp-Kyn metabolism, we sought to determine the mechanisms underlying the HFD-induced *Proteobacteria* overgrowth, a signature of gut dysbiosis. Our results showed that HFD decreased the colonic concentrations of bacteria-derived butyrate, the primary energy source for colonocytes, while upregulated long-chain and very long-chain fatty acid β-oxidation in colonocytes, triggering oxidative stress in colonic tissue that impaired mitochondrial function. Disrupted mitochondrial bioenergetics destabilized colonic epithelium hypoxia increasing luminal oxygen availability and caused metabolic reorientation in colonocytes towards glycolytic metabolism resulting in lactate release and the elevated nitrate synthesis. These provided additional respiratory electron donors or acceptors for *Proteobacteria* to thrive. Meanwhile, we noticed that dietary butyrate supplementation reversed the HFD-impaired mitochondrial bioenergetics and subsequent gut dysbiosis-induced dysregulation of Trp-Kyn metabolism. Collectively, our findings emphasized the causal role of the gut microbiota in diet-induced Trp metabolic disorders and shed light on the contribution of gut microbiota-colonocyte interactions in systemic metabolic homeostasis.

## Result

### HFD disturbed the serum metabolic profile

**Fig. 1:**
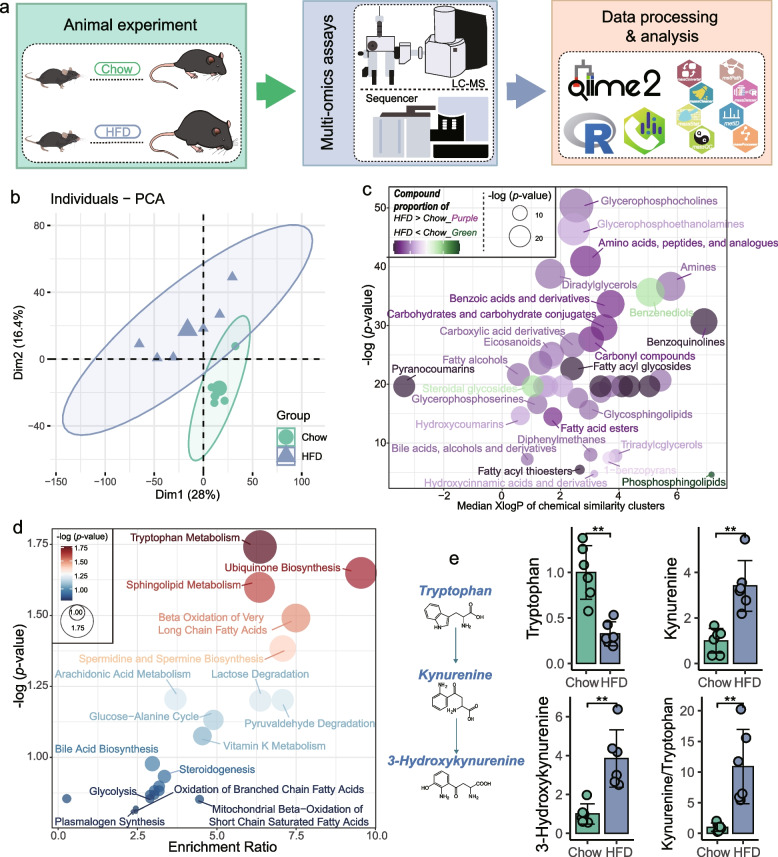
High-fat diet-disturbed tryptophan metabolism. **a** Experimental workflow started with animal dietary interventions and sample collection (*n* = 12 for each group). Multi-omics analysis was conducted to elucidate the mechanisms by which high-fat diet (HFD) affects metabolites. **b** Principal component analysis (PCA) score plot to assess serum metabolomic data comparing HFD-fed mice with standard diet-fed mice (Chow) (*n* = 6 for each group). Permutational multivariate analysis of variance (PERMANOVA) by Adonis was used to determine statistical significance. **c** Chemical similarity enrichment analysis (ChemRICH) clustering of 614 statistically HFD-altered serum metabolites by chemical similarity with *x*-axis of mediation logarithmic additive octanol–water partition coefficients (XlogP) and *y*-axis for sets statistical significance (Kolmogorov–Smirnov test, *p*-value < 0.05); the node size depicted total compound numbers for each cluster set and the node color scale the proportion of Chow-enriched vs. HFD-enriched metabolites. **d** Quantitative metabolite set enrichment analysis (qMSEA) based on 99 metabolite sets associated with human metabolic pathways identified the top 25 serum metabolic pathways significantly perturbed by HFD (*p*-value < 0.05). **e** HFD significantly upregulated the kynurenine (Kyn) metabolic pathway. Data are represented as mean ± SD and normalized to the Chow. In **e**, *p*-values were determined by independent samples *t*-test. NS not significant, * *p*-value ≤ 0.05, ** *p*-value ≤ 0.01, *** *p*-value ≤ 0.001. LC–MS liquid chromatography-mass spectrometry; Dim dimension

To uncover the impacts of the western diet on systemic metabolic homeostasis, 4-week-old mice were fed with HFD for 4 weeks to simulate a persistent western-style diet. We conducted untargeted metabolomics on serum from the standard diet (Chow)-fed and HFD-fed C57BL/6 mice at 8 weeks of age (Fig. 1a). Using UHPLC-HESI-HRMS-based untargeted metabolomics and Tidymass-based comprehensive computational framework, 3135 ion features were detected. We examined the metabolites globally using principal component analysis (PCA) and found distinct serum metabolic profiles due to dietary patterns (permutational multivariate analysis of variance (PERMANOVA) by Adonis, *p*-value = 0.003) (Fig. 1b). We then statistically evaluated the ion feature matrix and screened 614 significant features after removing redundant annotated metabolites (Wilcox test, *p*-value < 0.05). Chemical similarity enrichment analysis (ChemRICH) was performed to determine the categories of HFD-altered metabolites (Fig. 1c). For the chromatograms of the nodes, the purple color denoted that among the metabolites significantly altered between the Chow and HFD groups, the enriched metabolites in HFD exceeded those in Chow and/or reflected a more remarkable multiplicative change. Forty-one chemical classes were clustered, and HFD-altered metabolites are mainly involved in lipids and amino acids (Fig. 1c). To characterize the HFD-altered metabolites in the context of biological pathways, we carried out quantitative metabolite sets enrichment analysis (qMSEA) and found that HFD dramatically affected the systemic Trp metabolism (Fig. 1d). Notably, the serum Kyn pathway was significantly upregulated by HFD, as evidenced by enhanced 3-hydroxykynurenine (3-HK) and Kyn concentrations and Kyn/Trp ratio in serum (Fig. 1e). These results imply that persistent HFD disturbed serum Trp metabolism, characterized by upregulation of the Kyn pathway.

### Gut bacteria linked to serum Kyn concentration

**Fig. 2:**
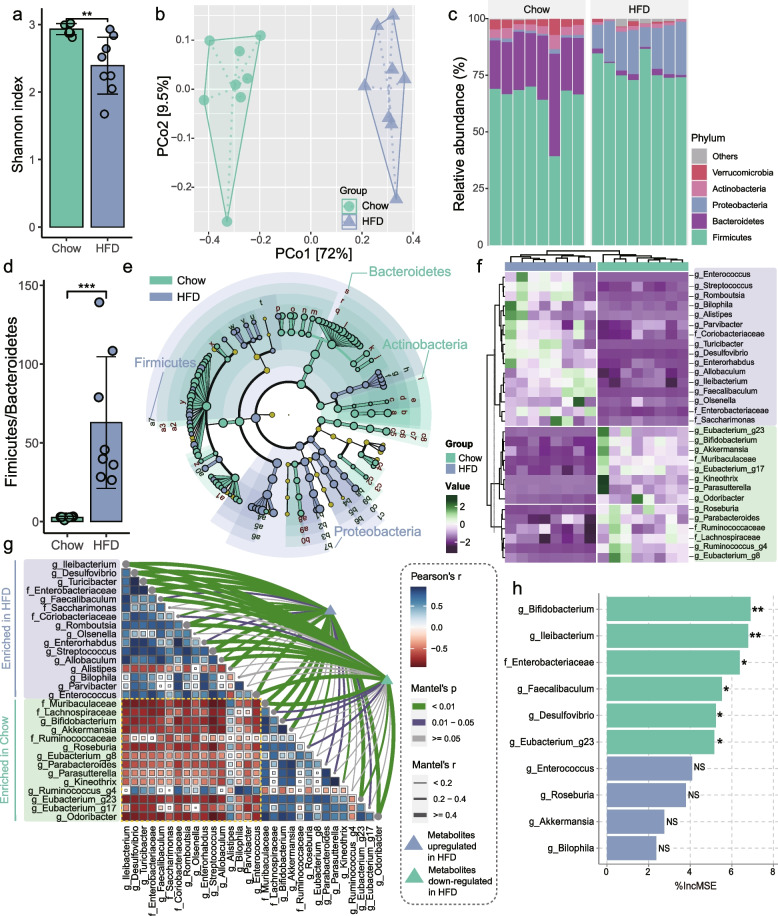
HFD-induced gut dysbiosis was highly correlated with serum metabolic profiles. **a** Gut bacterial alpha diversity was estimated by Shannon index (*n* = 8 for each group). **b** Principal coordinate analysis (PCoA) plot showing microbial compositional differences quantified by Bray–Curtis distance (PERMANOVA by Adonis). **c** Bar graph of bacterial abundance at the phylum level. **d** HFD enhanced the Firmicutes/Bacteroidetes ratio in the fecal microbiota. **e** Cladogram generated from linear discriminant analysis effect size (LEfSe) showing the most differentially enriched bacterial taxa in the colonic contents of Chow (green) or HFD (blue) mice (LDA value = 2.0; *p*-value < 0.05). **f** Heatmap of bacterial taxa abundance determined by LEfSe analysis. **g** Pairwise comparisons of HFD-altered bacterial taxa are shown, with a color gradient indicating Pearson’s correlation coefficients. HFD-altered serum metabolites (blue: HFD-enriched serum metabolites; green: serum metabolites downregulated in HFD-fed mice) were related to each bacterial taxon by *Mantel* tests. Edge width corresponds to *Mantel’s r* statistic for the corresponding distance correlations, and edge color indicates the statistical significance based on 999 permutations. **h** The top 10 bacterial biomarkers were identified by random forests regression of relative abundances of LEfSe-determined bacterial taxon against serum Kyn concentration. Statistical significance of selected bacterial biomarkers was assessed by permutation test (999 times). Data are represented as mean ± SD. In **a** and **d**, *p*-values were determined by independent samples *t*-test. NS not significant, * *p*-value ≤ 0.05, ** *p*-value ≤ 0.01, *** *p*-value ≤ 0.001. IncMSE increase in mean squared error

The intricate relationship between gut microbes and host metabolic homeostasis prompted us to investigate whether the observed changes in serum Trp metabolism were related to the response of gut bacteria to dietary patterns. In line with the previous reports [20], 16S ribosomal RNA (rRNA) gene amplicon sequencing of colonic contents revealed that persistent HFD significantly decreased the gut bacterial richness (Fig. 2a) and formed a distinct bacterial cluster relative to Chow mice (PERMANOVA by Adonis, *p*-value = 0.001) (Fig. 2b). Furthermore, we noticed that HFD mice exhibited expansion of the phylum *Proteobacteria* (Fig. 2c), a signature of gut dysbiosis, as well as an increment in the relative abundance of the phylum *Firmicutes* with respect to the phylum *Bacteroidetes* (Fig. 2c,d), which has been well established in the gut microbiota of obese patients [22]. To identify the HFD-altered bacterial taxa, the linear discriminant analysis (LDA) effect size (LEfSe) method was adopted (Fig. 2e,f). Our results indicated that butyrate-producing bacteria, including the genus *Roseburia*, *Eubacterium_g8*, *Eubacterium_g23*, and *Eubacterium_g17*, were decreased in HFD-fed mice, while persistent HFD thrived bacteria associated with intestinal inflammation, such as the genus *Bilophila* and *Desulfovibrio* and the family *Enterobacteriaceae* (Fig. 2e,f). Moreover, the expansion of *E. coli* in the intestine of HFD mice was further determined by *q*-PCR (Fig. S1). After correlating the HFD-altered bacterial taxa with the matrix of serum metabolites significantly disturbed in HFD mice, we found that the abundance of HFD-enriched bacterial taxa was strongly associated with changes in the serum metabolic profile (Fig. 2g). For example, the genus *Desulfovibrio* and the family *Enterobacteriaceae*, which were more abundant in HFD mice, were significantly associated with differential serum metabolites, whether up- or downregulated in HFD mice.

To further identify the key bacterial taxa associated with the Kyn pathway, we regressed the abundance of LEfSe-distinguished bacterial taxa against serum Kyn concentration using the random forests machine learning algorithm. Numerous studies have highlighted the association between *Proteobacteria* and metabolic diseases [23]. One underlying mechanism is that the expansion of *Proteobacteria* species (e.g., *Escherichia*, *Klebsiella*, and *Enterobacter* species) activates the gut mucosal immune, a process that relies on an active pro-inflammatory signaling cascade, disrupting intestinal homeostasis and contributing to local and systemic inflammation and metabolic dysfunction [23]. Similarly, we noticed that HFD-enriched lipopolysaccharide (LPS) producers (mainly belonging to the phylum *Proteobacteria* [24]), including the genus *Desulfovibrio* and the family *Enterobacteriaceae*, exhibited a significant positive correlation with serum Kyn concentration (Figs. 2h and S2a). These results revealed that HFD-induced gut dysbiosis, characterized by expansion of the phylum *Proteobacteria*, was strongly associated with upregulation of the Kyn pathway in serum.

### HFD disrupted the peripheral Kyn pathway in a gut microbiota-dependent manner

**Fig. 3:**
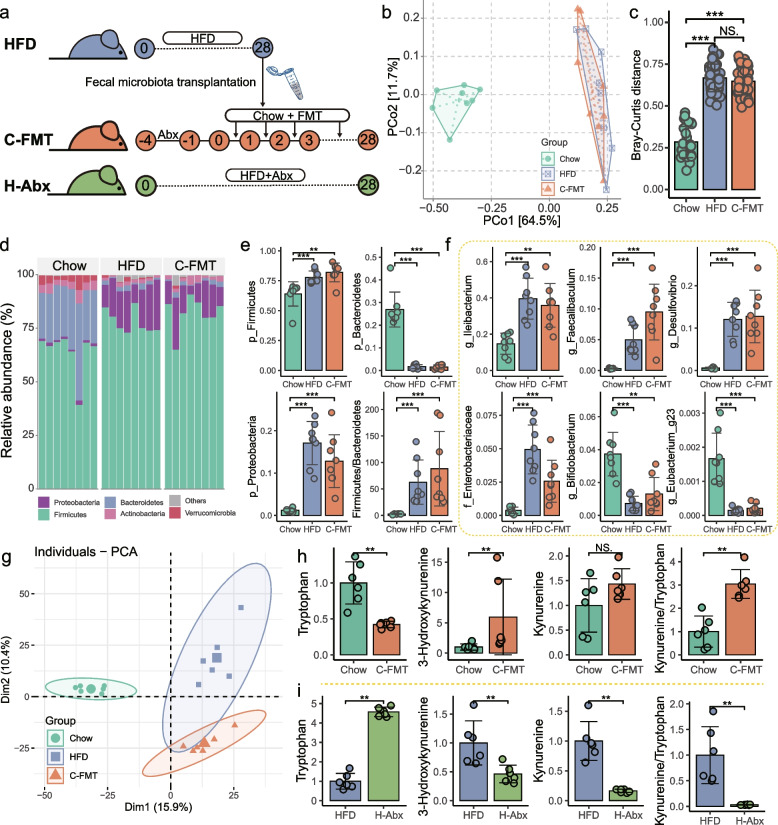
The causal role of gut microbiota in mediating HFD-induced dysregulation of peripheral tryptophan-kynurenine metabolism. **a** Fecal pellets were collected from HFD mice at 8 weeks and then used to perform fecal microbiota transplantation (FMT). Standard diet-fed mice were treated with an antibiotic cocktail for 3 days, followed by FMT lasting 4 weeks (C-FMT). HFD-fed mice were concurrently treated with an antibiotic cocktail for 4 weeks (H-Abx) to further verify the critical role of gut microbiota in diet-mediated disruption of tryptophan (Trp)-kynurenine (Kyn) metabolism. **b,c** PCoA based on Bray–Curtis distance showing the similarity of microbiota composition in mice after FMT to that of the donor (*n* = 8 for each group). **d** Bar graph of bacterial abundance at the phylum level. **e–f** C-FMT mice exhibited a taxonomic profile of gut bacteria more comparable to that of HFD mice. **g** PCA was performed to assess the serum metabolic profile in standard diet-fed mice after FMT donated by HFD mice (*n* = 6 for each group; PERMANOVA by Adonis). **h** FMT donated by HFD mice enhanced the peripheral Trp-Kyn pathway in standard diet-fed mice. **i** Clearing gut bacteria by antibiotic cocktail abolished HFD-induced dysregulation of peripheral Trp-Kyn metabolism. Data are represented as mean ± SD. In **h**, data are normalized to Chow after FMT. In **i**, data are normalized to HFD after antibiotic cocktail treatment. In **c**,** e**, and** f**, *p*-values were determined by one-way analysis of variance (ANOVA) with Dunnett’s multiple comparison test. In **h,i**, statistical significance was assessed by independent samples *t*-test. NS not significant, * *p*-value ≤ 0.05, ** *p*-value ≤ 0.01, *** *p*-value ≤ 0.001

In view of the link between gut bacteria and the Kyn concentration in serum, we further explored the causality of HFD-induced gut dysbiosis in the dysfunction of the peripheral Trp-Kyn pathway. We transplanted the fecal microbiota of HFD-fed mice into standard diet-fed mice (C-FMT) and subsequently examined Kyn pathway metabolites in the serum of C-FMT mice (Fig. 3a). The efficiency of microbial colonization is closely related to the availability of niches in the context [25]. Therefore, to improve the efficiency of fecal microbiota transplantation (FMT), mice were treated with an antibiotic cocktail (Abx) for 3 days prior to FMT to eliminate the commensal gut flora [26]. The remaining bacteria abundance in the feces after Abx treatment was measured by *q*-PCR (Fig. S3a). Our results showed that oral gavage of Abx for 3 days eliminated more than 80% of the gut native microbiota (Fig. S3b-c). After 4 weeks of continuous daily FMT, the microbial composition of the C-FMT mice was more similar to that of the HFD mice compared to the Chow mice (Fig. 3b,c). At the phylum level, C-FMT mice displayed an increased abundance of *Proteobacteria* and a higher *Firmicutes*/*Bacteroidetes* ratio compared to Chow mice (Fig. 3d,e). Meanwhile, the abundance pattern of key bacterial taxa identified by microbe-metabolite association studies (Figs. 2g–h and S2) in C-FMT mice was consistent with that of HFD mice (Fig. 3f). These results indicate that standard diet-fed mice receiving FMT significantly remodeled their gut microbiota to more closely resemble the bacterial composition of HFD mice.

Next, we examined the changes in the serum metabolism of C-FMT mice by performing untargeted metabolomics and found that FMT donated by HFD mice dramatically altered the metabolic profile of standard diet-fed mice to be more comparable to that of HFD mice (Fig. 3g). We further checked the Trp-Kyn metabolism in serum and found a depletion of Trp and an increment in both 3-HK concentration and Kyn/Trp ratio in C-FMT mice (Fig. 3h). Based on the above results, we confirmed that the dysregulation of peripheral Trp-Kyn metabolism in HFD mice is, at least in part, attributed to the diet-altered gut microbiota. Given the intimate relationship between dietary composition and individual metabolic profiles, we sought to determine whether HFD could direct systemic Trp-Kyn metabolism independently of gut microbiota. Pseudo germ-free mice were generated by administering antibiotic combinations to HFD-fed mice (H-Abx) to further confirm the role of gut microbiota in HFD-induced dysregulation of peripheral Trp-Kyn metabolism (Fig. 3a). As shown in Fig. 3i, H-Abx mice exhibited decreased activity of the Kyn pathway in serum compared to HFD mice, suggesting that gut microbiota played a determining role in mediating the dysfunctional Trp-Kyn metabolism caused by HFD. Collectively, these results highlighted that HFD disrupted peripheral Trp-Kyn metabolism in a gut microbiota-dependent manner.

### Inhibition of IDO1 in colonocytes attenuated gut dysbiosis-induced serum Kyn accumulation

**Fig. 4:**
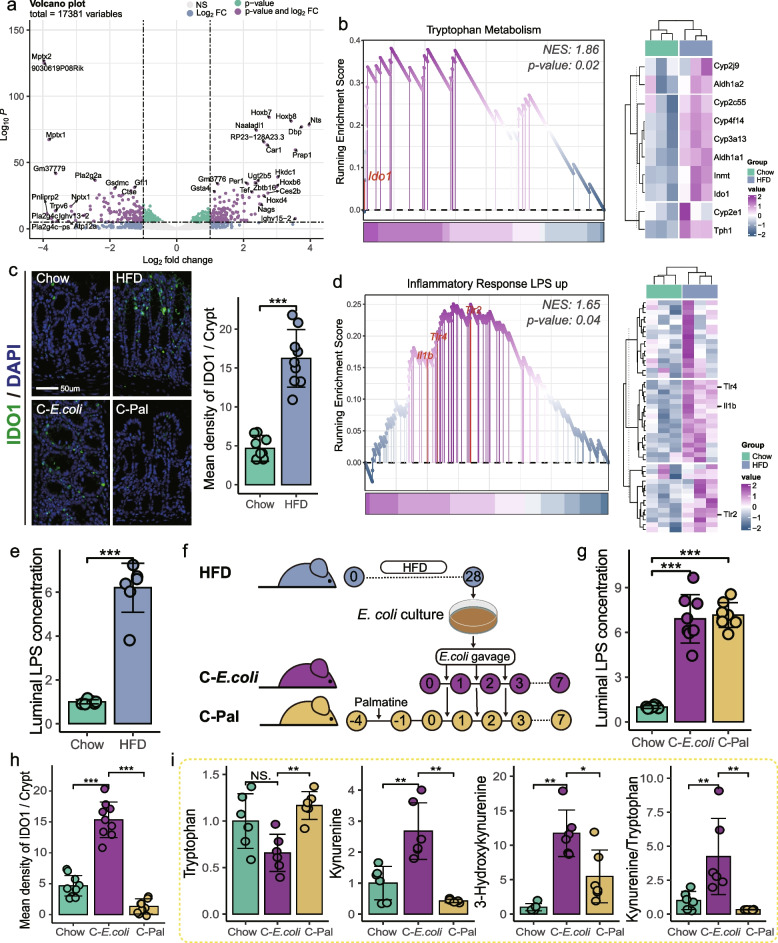
Hyperactivated indoleamine 2,3-dioxygenase 1 in colonocytes mediated the gut dysbiosis-induced dysregulation of peripheral Trp-Kyn metabolism. **a** Volcano plot showing the HFD-altered transcriptomes in colonocytes (*n* = 3 for each group). **b** Gene set enrichment analysis (GSEA) showing enrichment of the Trp metabolism gene sets (left) and heatmap of involved genes (right) (*n* = 3 for each group). **c** Fluorescent immunostaining of indoleamine 2,3-dioxygenase 1 (IDO1) (green) in mouse colon sections. Nuclei were counterstained with DAPI (blue) (*n* = 9 slices from 3 mice). **d** Enriched gene sets of lipopolysaccharide (LPS)-induced inflammation (left) and the heatmap of involved genes (right) (*n* = 3 for each group). **e** HFD enhanced the LPS concentration in the colonic contents (*n* = 8 for each group). **f** *Escherichia coli* (*E. coli)* indicator strains were isolated from the feces of HFD mice and enriched in vitro for bacterial transplantation to confirm the causal role of *Proteobacteria* expansion in HFD-induced dysregulation of Kyn pathway (C-*E.coli*). To determine the central role of colonic IDO1 in *E.coli-*mediated upregulation of the Kyn pathway, mice were administrated with palmatine to eliminate colonic IDO1 before receiving *E.coli* (C-Pal). **g E. coli** transplantation enhanced LPS concentration in the colonic contents (*n* = 8 for each group). **h** IDO1 activity in the colon (*n* = 9 slices from 3 mice). **i** *E. coli* transplantation upregulated the serum Kyn pathway, which was reversed by palmatine pre-treatment (*n* = 6 for each group). Data are represented as mean ± SD. In **e**, **g**, and **i**, data are normalized to Chow. In **c** and** e**, *p*-values were determined by independent samples *t*-test*.* In **g**–**i**, statistical significance was assessed by ANOVA with Dunnett’s multiple comparison test. NS not significant, * *p*-value ≤ 0.05, ** *p*-value ≤ 0.01, *** *p*-value ≤ 0.001

Given the central role of IDO1 in regulating the Trp-Kyn pathway [2], we hypothesized that the HFD-induced gut dysbiosis might hyperactivate IDO1 in the colon to increase serum Kyn concentrations. To investigate the effect of HFD on the interaction between gut microbiota and colonocytes, we compared the gene expression profiles of colonic tissue between Chow and HFD groups using RNA sequencing. There were significant differences in the transcriptomes of colonic tissues between Chow and HFD mice (PERMANOVA by Adonis, *p*-value = 0.016) (Fig. S4a). We identified 401 (absolute log_2_-fold change (log_2_FC) > 1, *p*-value < 0.05) out of 17,381 gene transcripts that were significantly associated with HFD (Fig. 4a). Gene set enrichment analysis (GSEA) indicated that “tryptophan metabolism” pathway was significantly upregulated in colonic tissue of HFD mice compared to that in Chow mice (*p*-value = 0.02), characterized by high expression of the IDO1 gene (Fig. 4b). To further confirm the expression of IDO1 in colon tissue, we performed immunofluorescence staining and found a significant increase of IDO1 in the colon of HFD mice (*t*-test, *p*-value < 0.001) (Fig. 4c). These results revealed that persistent HFD upregulated IDO1 in colonocytes. Given the stimulatory effect of pro-inflammatory cytokines on IDO1, we next examined the immune response in colon. Consistent with the result of *Proteobacteria* expansion in the colonic microbiota (Fig. 2c, e), bacterial LPS (mainly derived from the phylum *Proteobacteria* [24]) mediated inflammatory response was significantly activated in colonic tissue (*p*-value = 0.04) (Fig. 4d), which was also validated by the increased concentration of LPS in colonic contents (Fig. 4e).

To verify the causative role of *Proteobacteria* expansion in colonic IDO1 upregulation and subsequently disrupted serum Trp-Kyn metabolism, we isolated *E. coli* indicator strains from the feces of HFD mice and enriched them in vitro (> 1 × 10^8^ CFU/ml) for bacterial transplantation to standard diet-fed mice (Fig. 4f). Oral gavage of *E.coli* for 3 days successfully established *E.coli* in the colonic lumen of mice on a standard diet (C-*E.coli*), as evidenced by the increased abundance of *E.coli* in the feces of mice on day 7 (Figs. 4f and S5a). As expected, *E. coli* expansion significantly increased the concentration of LPS in the intestinal lumen (post hoc Dunnett’s test, *p*-value < 0.001) (Fig. 4g) and the expression of IOD1 in colonic tissue (post hoc Dunnett’s test, *p*-value < 0.001) (Fig. 4c, h), accompanied by an upregulation of the Kyn pathway in serum (Fig. 4i). These results emphasized the initiating role of *Proteobacteria* expansion in HFD-induced disturbances of Trp-Kyn metabolism. We further administrated standard diet-fed mice with palmatine, an irreversible IDO1 inhibitor with extremely low oral bioavailability [27, 28], to investigate the dominant effect of colonic IDO1 on peripheral Trp-Kyn metabolism (C-Pal). Several studies have suggested an inhibitory effect of palmatine on gram-negative bacteria such as *E. coli* [29]. Therefore, palmatine was discontinued when mice received *E. coli* indicator strains isolated from HFD mice (Fig. 4f). Similar to C-*E.coli* mice, the aftereffects of palmatine did not affect the colonization of *E. coli* in the colon of C-Pal mice (Fig. S5a). Meanwhile, our results showed that oral palmatine significantly inhibited colonic IDO1 (post hoc Dunnett’s test, *p*-value < 0.001) (Fig. 4c, h) and suppressed the serum Kyn pathway in C-Pal mice (Fig. 4i), even though the concentration of LPS in the colonic contents of these mice was higher than that of Chow mice (Fig. 4g). According to the above results, we concluded that the overgrowth of *Proteobacteria*, which triggered the upregulation of IDO1 in the colon, played a determinant role in the HFD-induced dysregulation of peripheral Trp-Kyn metabolism.

### HFD-disrupted mitochondria in colonocytes promoting the accessibility of host-derived respiratory substrates to Proteobacteria

**Fig. 5:**
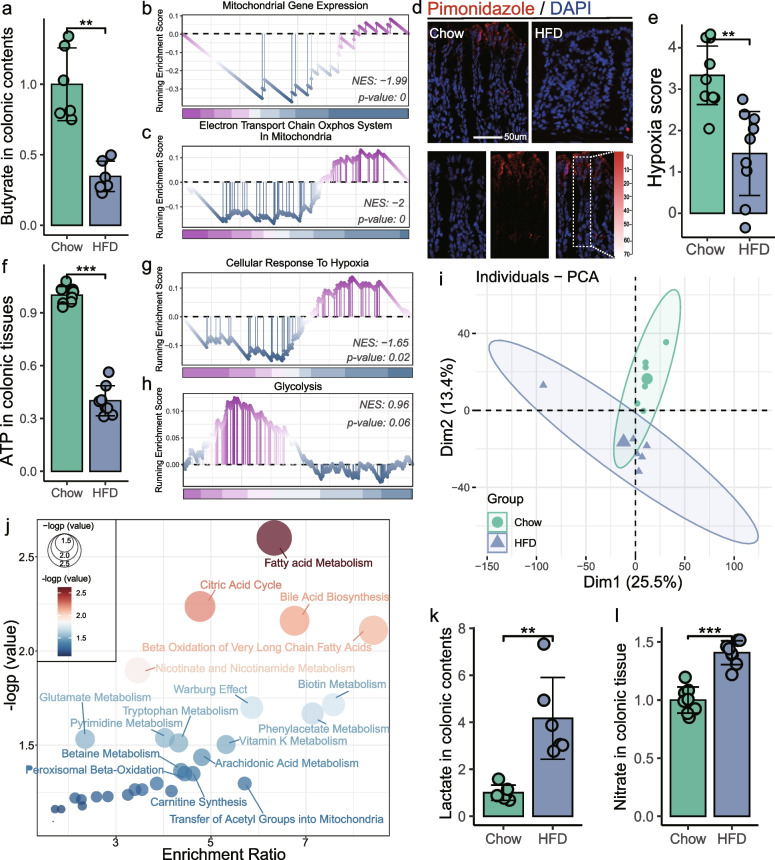
HFD disrupted mitochondrial function and caused metabolic reorientation in colonocytes. **a** HFD reduced butyrate concentrations in colonic contents (*n* = 6 for each group). HFD inhibited mitochondrial gene expression (**b**) and mitochondrial oxidative phosphorylation (**c**). **d** Binding of pimonidazole (red) was used to qualify the oxygen gradient in the colon. Nuclei were counterstained with DAPI (blue). **e** Pimonidazole staining was quantified by scoring blinded sections of the colon (*n* = 9 slices from 3 mice). **f** Adenosine triphosphate (ATP) concentrations in colonic tissue (*n* = 8 for each group). **g** HFD downregulated HIF-α-mediated epithelial hypoxia in colonic tissue. **h** HFD induced a shift in the metabolism of short-chain fatty acids (SCFAs) towards glycolysis in colonic tissue. **i** PCA score plot for assessing colonic metabolomic data comparing HFD mice with Chow mice (*n* = 6 for each group; PERMANOVA by Adonis). **j** qMSEA method was adopted to identify the colonic metabolic pathways perturbed by HFD, and the top 30 were shown. HFD enhanced the lactate in colonic contents (**k**,* n* = 6) and nitrate in colonic tissue (**l**, *n* = 8). Data are represented as mean ± SD. In **a**, **f**, **k**, and **l**, data are normalized to Chow, and statistical significance was assessed by independent samples *t*-test. In **e**, the *p*-value was determined by the Wilcoxon test. NS not significant, * *p*-value ≤ 0.05, ** *p*-value ≤ 0.01, *** *p*-value ≤ 0.001. NES normalized enrichment scores

Colonocyte metabolism is critical in shaping the colonic microbiota [30]. One mechanism is that the host limits the availability of oxygen and nitrate in the colonic lumen, which is favorable for the growth of obligate anaerobes that specialize in fermentation [7]. In turn, butyrate derived from obligate anaerobes activates peroxisome proliferator-activated receptor-γ (PPAR-γ) [26], which promotes mitochondrial β-oxidation of short-chain fatty acids (SCFAs) in colonocytes to generate adenosine triphosphate (ATP) for maintaining energy metabolism [26, 31]. Given the dramatic shift in the transcriptional profile of colonic tissue (Figs. 4a and S4a), we therefore hypothesized that the HFD-induced colonocyte dysfunction might contribute to driving the overgrowth of *Proteobacteria*. Consistent with the reduction in the abundance of butyrate-producing bacteria (Fig. 2e,f), persistent HFD significantly decreased the concentration of colonic butyrate (*t*-test, *p*-value < 0.01) (Fig. 5a), which provides more than 70% of the energy for colonic epithelial cells [32]. Meanwhile, we noticed that long-chain and very long-chain fatty acid metabolic process was increased in the colonocytes of HFD mice (Fig. S6a-b). Previous studies have shown that mitochondrial β-oxidation of long-chain fatty acids increased the ratio between electrons entering the respiratory chain via FADH2 or NADH (the longer the fatty acid, the higher the ratio), leading to higher levels of reactive oxygen species (ROS) generation that impairs mitochondrial function [33]. Consistent with the increased oxidative stress response in colonocytes (Fig. S6c), HFD mice showed impaired mitochondrial activity in colonic tissue, as indicated by the reduced mitochondrial gene expression (Fig. 5b) and oxidative phosphorylation (Fig. 5c), as well as the reduced ATP levels (Fig. 5f).

In the colonocytes, oxidative phosphorylation in mitochondria consumes oxygen to maintain colonic epithelial hypoxia, which maintains the anaerobic properties of the colonic lumen to promote the dominance of obligate anaerobes while inhibiting the growth of facultative anaerobes such as *Enterobacteriaceae* [30]. To investigate the oxygenation of colonic epithelium in case of HFD-impaired mitochondrial bioenergetics, we visualized the colonic epithelial hypoxia using the exogenous hypoxic marker pimonidazole (Fig. 5d). Pimonidazole staining revealed that colonic epithelial hypoxia was eliminated in mice receiving HFD (Fig. 5e), indicating increased availability of luminal oxygen, which is the main factor regulating *Proteobacteria* growth in the intestine [23]. The increased oxygen concentration disrupted physiological hypoxia in colonocytes (Fig. 5g), which is crucial for the adaptation of cell metabolism and various processes such as barrier function and immunity [34]. An important immunological feature of the colon is the highly glycosylated and hydrated mucus layer [35], which provides structural scaffolding and a nutrient source for gut microbiota such as *Akkermansia* [36]. Consistent with the increased epithelial oxygenation, sustained HFD inhibited mucus production in colon, as evidenced by immunofluorescence of intestinal secretory mucin-2 (MUC2) (Fig. S7a).

The impaired mitochondrial bioenergetics leads to a metabolic reorientation towards glycolytic metabolism in colonocytes, even in the presence of oxygen (Fig. 5h), characterized by high lactate release, low oxygen consumption, and increased nitrate synthesis [30]. Untargeted metabolomics was performed to examine colonic metabolic reprogramming in HFD mice. The PCA result showed a distinct metabolic profile between Chow and HFD mice (PERMANOVA by Adonis, *p*-value = 0.006) (Fig. 5i). Consistent with colon RNA sequencing results (Figs. 4b, 5g,h, and S6a-b), qMSEA revealed that the HFD-altered colonic metabolites (*n* = 554, *p*-value < 0.05) were involved in the fatty acid metabolism, β-oxidation of very long-chain fatty acids, Trp metabolism, and the Warburg effect, a hallmark of aerobic glycolysis (Fig. 5j). At the same time, we detected the significant increase in lactate concentration in the colonic contents (*t*-test, *p*-value < 0.01) (Fig. 5k) and nitrate in the colonic tissue of HFD mice (*t*-test, *p*-value < 0.001) (Fig. 5l). Host-derived nitrate could be used as an electron acceptor by several members of the *Proteobacteria* (e.g., *Enterobacteriaceae*) for anaerobic respiration to generate ATP [30]. Bacteria use the redox reaction with the greatest free energy to prevail, determining which metabolic groups of bacteria can dominate microbial communities in habitats. These results suggest that the HFD-induced mitochondria impairment and following metabolic reorientation in colonocytes enhanced the accessibility of respiratory substrates for *Proteobacteria*, which represented a potential mechanism underlying the HFD-induced gut dysbiosis.

### Remodeling of colonic mitochondrial bioenergetics alleviated the gut dysbiosis-induced dysregulation of Trp-Kyn metabolism

**Fig. 6:**
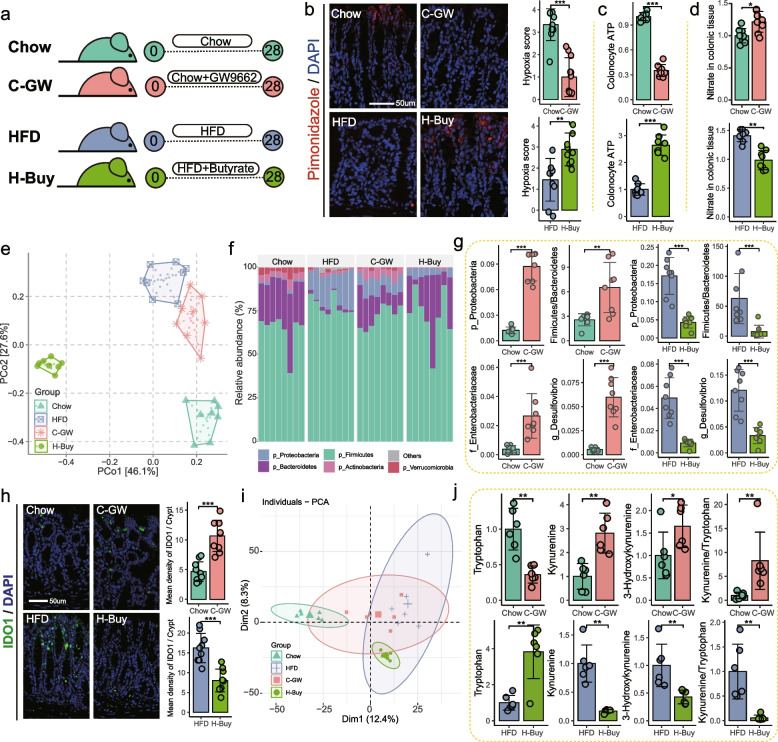
Colonic mitochondrial dysfunction-induced gut dysbiosis disturbed systemic Trp-Kyn metabolism. **a** Standard diet-fed mice were administrated with GW9662 to examine the relationship between colonic mitochondrial dysfunction and Trp metabolism (C-GW). To confirm the beneficial effect of remodeling colonic mitochondrial bioenergetics on systemic Trp-Kyn metabolism, butyrate was provided to HFD-fed mice in drinking water (H-Buy). **b** Colonic epithelial oxygenation was determined by pimonidazole (red). Nuclei were counterstained with DAPI (blue) (*n* = 9 slices from 3 mice). Concentrations of ATP (**c**) and nitrate (**d**) in colonic tissue (*n* = 8 for each group). **e** PCoA showed the similarity of gut microbiota determined by 16S profiling (*n* = 8 for each group; PERMANOVA by Adonis). **f** Relative abundance of the bacterial phylum. **g** Relative abundance of phylum *Proteobacteria*, family *Enterobacteriaceae*, and genus *Desulfovibrio* and the ratio of *Firmicutes*/*Bacteroidetes*. **h** Fluorescent immunostaining of IDO1 (green) in mouse colon sections. Nuclei were counterstained with DAPI (blue) (*n* = 9 slices from 3 mice). **i** PCA score plot showing serum metabolic profiles (*n* = 6 for each group; PERMANOVA by Adonis). **j** Serum metabolites involved in the Kyn pathway (*n* = 6 for each group). Data are represented as mean ± SD. In **c**, **d**, and **j**, data are normalized to Chow or HFD respectively. In **b**, the *p*-value was determined by the Wilcoxon test. Other statistical procedures were performed by independent samples *t*-test. NS not significant, * *p*-value ≤ 0.05, ** *p*-value ≤ 0.01, *** *p*-value ≤ 0.001

To confirm the causality between colonocyte mitochondrial dysfunction and *Proteobacteria* overgrowth, the standard diet-fed mice were treated with the PPAR-γ antagonist GW9662 to inhibit mitochondrial oxidative phosphorylation in colonocytes (C-GW) (Fig. 6a). In line with the observations in HFD mice, C-GW mice showed reduced concentrations of ATP (Fig. 6c) and increased epithelial oxygenation (Fig. 6b) and nitrate production (Fig. 6d) in colonic tissue. Furthermore, 16S rRNA profiling revealed that GW9662-inhibited colonic mitochondrial bioenergetics altered the gut bacterial composition, which was more similar to that of HFD mice compared to Chow mice (Fig. 6e), characterized by the enhanced *Firmicutes*/*Bacteroidetes* ratio and abundance of the phylum *Proteobacteria*, as well as overgrowth of the genus *Desulfovibrio* and family *Enterobacteriaceae* (Fig. 6f,g). Meanwhile, upregulated colonic IDO1 (Fig. 6h) and a disturbed serum metabolic profile characterized by enhanced Kyn pathway (Fig. 6i,j) were also observed in C-GW mice, suggesting a dominant role of colonocyte mitochondrial dysfunction in gut dysbiosis and subsequent Trp-Kyn metabolic dysregulation.

Given the reduction of bacteria-fermented butyrate in colonic contents (Fig. 5a), which resulted in a shift in colonocyte energy metabolism towards β-oxidation of long- and very long-chain fatty acids (Figs. 5j and S6a-b), we wanted to investigate whether remodeling of colonocyte mitochondrial bioenergetics by butyrate supplementation could alleviate HFD-induced dysregulation of Trp-Kyn metabolism. To this end, HFD-fed mice were supplied with butyrate in the drinking water for 4 weeks (H-Buy) (Fig. 6a). In mice receiving HFD, butyrate supplementation restored colonic epithelial hypoxia (Fig. 6b) and mitochondrial activity (Fig. 6c), and reduced nitrate production (Fig. 6d) in colonic tissue. Consistent with the reduction in respiratory electron acceptors utilized by *Proteobacteria*, H-Buy mice exhibited a distinct bacterial profile compared to HFD mice (PERMANOVA by Adonis, *p*-value = 0.001) (Fig. 6e,f), in particular a reduced abundance of LPS-producing bacteria (Fig. 6g), indicating a beneficial effect of butyrate on the gut microbiota. Furthermore, H-Buy mice exhibited lower colonic IDO1 expression (Fig. 6h) and downregulated Kyn pathway relative to HFD mice (Fig. 6j). Taken together, these results re-emphasize the critical nature of the interactions between the gut microbiota and colonocytes for the maintenance of systemic Trp-Kyn metabolic homeostasis.

## Discussion

A cross-sectional study indicated the accompaniment of the Western-style diet with aberrant Trp metabolism, characterized by upregulated Kyn pathway and exhausted circulating Trp [37]. In light of recent evidence, overexpressed IDO1 in adipocytes has been identified as the cause of excessive Kyn in rodent models with HFD [11]. Similarly, most previous studies have focused on the tissues that specifically express IDO1, such as the liver, muscle, and adipocytes [1, 2, 6], while the contribution of the gut microbiota in the diet-associated Trp metabolic disorder has been less investigated. Accumulating evidence points to the gut microbiota as a central mediator in diet-related chronic diseases and metabolic syndrome [19]. In the present study, we demonstrated that HFD-impaired colonocyte mitochondrial bioenergetics promoted the expansion of colonic *Proteobacteria*, which in turn upregulated the inflammatory response and subsequent IDO1 expression in colonocytes, ultimately dysregulating systematic Trp-Kyn metabolism. Below, we discussed how these findings broaden our knowledge of the central role and underlying mechanisms of gut microbiota-colonocyte interactions in diet-disrupted Trp metabolism.

Generally, less than 1% of Trp obtained through the diet is utilized for protein synthesis, and over 95% is metabolized along the Kyn pathway [2]. The best-studied rate-limiting enzymes for tissue-specific expression in the Kyn pathway are IDO1 and tryptophan-2,3-dioxygenase (TDO). TDO is not expressed in the gut and will not be discussed here. Furthermore, the expression pattern of IDO1 is more pathologically relevant. For example, colon tissue from patients with inflammatory bowel disease and colorectal cancer is often accompanied by high expression of IDO1 [1, 38]. In some cases, Kyn is considered to be an immunosuppressive agent, partly due to the accompaniment of increased Kyn following inflammation [39]. However, researchers also found that injection of exogenous Kyn into mice did not alleviate the inflammatory microenvironment, in contrast, impaired glucose tolerance and aggravated insulin resistance [11], suggesting a negative effect of increased circulating Kyn on individual health. These findings suggest a complex role for Kyn and its metabolites in diverse pathological conditions. Nevertheless, the involvement of the aberrant Trp-Kyn pathway in human disease is well accepted. The central nervous system receives approximately 60% of Kyn from the circulation; therefore, functional hyperactivation of the peripheral Kyn pathway, commonly triggered by inflammation, may initiate or facilitate central nervous system disorders [40]. Kyn is usually hydroxylated to 3-HK and then further converted to quin, which exerts neurotoxicity [41]. Despite the recognized negative impact of abnormal Kyn metabolism on human health, the source of the aberrant increased Kyn in the periphery is mainly elusive.

**Fig. 7:**
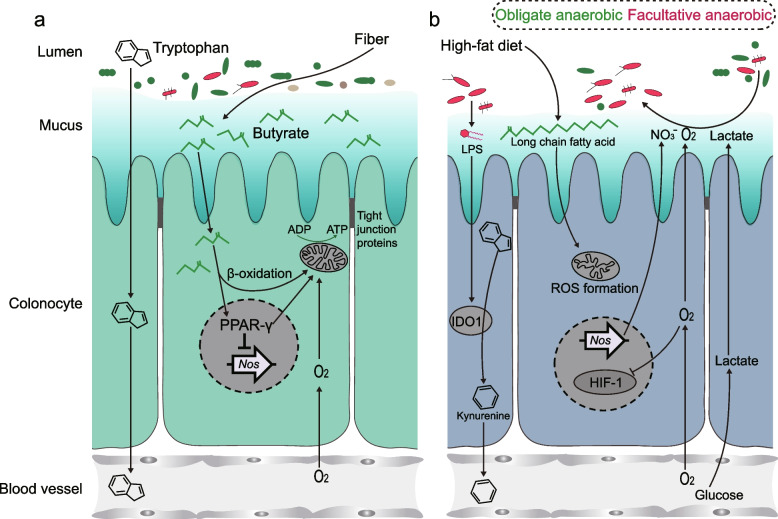
Schematic diagram of the mechanism underlying HFD-dysregulated peripheral Trp-Kyn metabolism. **a** In the healthy gut, the gut microbiota is dominated by obligate anaerobic bacteria that convert fiber into fermentation products, such as butyrate, to maintain colonocyte metabolism. **b** Sustained HFD reduced colonic butyrate concentrations and enhanced β-oxidation of long- and very long-chain fatty acids in colonic cells, which impaired mitochondrial bioenergetics and caused a metabolic reprogramming of colonic cells, allowing *Proteobacteria* to thrive. Bacteria-derived LPS stimulated colonic immune responses to upregulate the IDO1-mediated Kyn pathway

An increase in the consumption of Western-type diets, including ultra-processed foods and convenience products, has been linked to the development of non-communicable diseases, which nowadays cause more than 80% of deaths in Western societies [15, 42]. The long-term Western-style diet can disrupt physiological homeostasis through pathological transformations in lipids, induction of metabolic syndrome, and hyperactivation of the immune system [15]. Growing research indicates that tissue-specific and systemic immune responses are highly integrated with metabolic regulation, which is regarded as central to the maintenance of organismal homeostasis [15]. Low-grade chronic inflammation has been thought to contribute to IDO1 activation [9]. Meanwhile, over-activation of the Kyn pathway has been implicated in the development of pathologies in inflammatory situations [2]. Accumulating evidence reveals that diet-related diseases have been linked to dysbiosis of the gut microbiota [19]. The intestinal commensal microbiota is mainly located in the human colonic segment, a variable and complex system that requires sustained barriers and modulatory mechanisms to maintain host-microbe interactions, tissue and immune homeostasis, and overall individual physiology [15, 43]. Within the range of factors that influence the gut microbiota, food intake and dietary habits exert the dominant effect on microbial composition and function [44]. Persistent HFD for mice caused dramatic gut dysbiosis, characterized by the overgrowth of pathobionts (phylum *Proteobacteria*) and the reduction of host-beneficial bacteria (Fig. 2c–f). The development of techniques to study microbiota-host interactions, such as germ-free mice and FMT [45], has provided us with tools to investigate the causal role of gut microbiota in disease progression. Standard diet-fed mice receiving FMT from HFD mice exhibited disruption in Trp-Kyn metabolism (Fig. 3g,h), which was abolished in HFD mice by administration of an antibiotic cocktail (Fig. 3i). These results provide the first evidence for the central role of gut microbiota in the HFD-induced dysregulation of Trp-Kyn metabolism. Mechanistically, the dysbiotic microbiota stimulates the intestinal mucosal immune system through several mechanisms, such as modified signaling via the Toll-like receptors (TLRs) (Fig. 4d,e) [46], accompanied by reduced mucus release into the lumen (Fig. S7a) [35]. These factors, in turn, lead to disruption of barrier integrity and dysfunction of intestinal immune homeostasis, which upregulated the expression of colonic IDO1 to increase the peripheral Kyn concentration (Figs. 4b,c and 7b).

It is well established in animal models that colonocyte metabolism acts as a control switch, bridging the transition between homeostasis and dysbiosis in gut microbial communities (Fig. 7a) [30]. Cumulative evidence supports that HFD disrupts host control over the microbial community, which causes structural shifts in the commensal gut bacteria [17]. Our results showed that persistent HFD enhanced *Firmicutes*/*Bacteroidetes* ratio (Fig. 2d), a feature of the gut microbiota in obese patients [22]. Colonic obligate anaerobic bacteria convert undigested dietary fiber to fermentation products that are directly absorbed into colonocytes and then oxidated in the mitochondria resulting in high epithelial oxygen consumption to maintain the epithelium in a state of physiological hypoxia (Fig. 5d) [26, 30]. Among the bacteria-fermented metabolites, butyrate was the primary energy source (over 70%) for colonocytes and activated PPAR-γ to reinforce mitochondrial β-oxidation and oxygen consumption [26]. Low levels of intracellular oxygen concentration inhibit prolyl-hydroxylases and factor-inhibiting HIF to maintain HIF-1α and allow its translocation to the nucleus, in relation to the regulation of metabolism and barrier function in intestinal epithelial cells (Figs. 5j and S7a) [47]. Furthermore, epithelial hypoxia limits the diffusion of oxygen into the colonic lumen, which supports an anaerobic environment in the colonic lumen and drives the dominance of beneficial obligate anaerobic bacteria in the gut microbiota (Fig. 7a) [7]. These insights indicate the critical role of mitochondrial bioenergetics in colonocyte metabolism shaping the gut microbiota.

In the present study, we noticed that persistent HFD increased the β-oxidation of long-chain and very long-chain fatty acids in colonocytes (Figs. 5j and S6a-b). Oxygen radical formation accompanies with β-oxidation in mitochondria and is determined by the substrate for mitochondrial respiration [48, 49]. Dave proposes a kinetic model in which the ratio between electrons entering the respiratory chain via FADH2 or NADH (the F/N ratio) is a crucial determinant of ROS formation in mitochondria [33]. During glucose oxidation, the ratio is low (0.2), while long-chain fatty acids, such as palmitic acid (16 C atoms), instead result in an F/N ratio (15/31) of 0.48. Oxidative stress induced by ROS accumulation impairs mitochondrial bioenergetics in the colonic epithelium (Fig. 5b,c, f) [50]. As mitochondrial oxygen consumption maintains physiological hypoxia at the colonic surface, persistent HFD impaired mitochondrial functions to increase colonic epithelial oxygenation (Fig. 5d), which increased oxygen availability in the colonic lumen to promote the expansion of *Proteobacteria* via aerobic respiration. Meanwhile, the reduced mitochondrial activity in colon epithelial cells induced the release of nitrate (Fig. 5l), which is also available for *Proteobacteria* as electron acceptors [8]. Notably, persistent HFD enriched the colonic *Desulfovibrio* (Fig. 2e,f), which is typically grown anaerobically and can tolerate low levels of exposure to air [51]. An important characteristic of *Desulfovibrio* is its ability to utilize lactate as an electron donor for respiration [52]. The impaired colonic mitochondrial function causes a shift from oxidative phosphorylation to anaerobic glycolysis in colonocytes, characterized by low oxygen consumption and increased lactate production and secretion (Figs. 5k and 7b) [34], supporting *Desulfovibrio* expansion. In addition to producing LPS, *Desulfovibrio* is able to perform dissimilatory sulfate reduction by utilizing sulfate to produce hydrogen sulfide (H_2_S) [53]. Interestingly, other H_2_S producers, including *Streptococcus* and *Bilophila*, were also enriched in HFD mice (Fig. 2f). High levels of H_2_S are reported to be cytotoxic, which may contribute to colonic inflammation. The causality between the impaired mitochondrial bioenergetics in colonocytes and the HFD-induced dysregulation of peripheral Trp-Kyn metabolism was further confirmed by inhibiting colonic mitochondrial β-oxidation in standard diet-fed mice using GW9662, a PPAR-γ antagonist (Fig. 6).

An early understanding of the mechanisms that destabilize the gut microbiota was gained by investigating the consequences of antibiotic treatment, which disrupts colonic epithelial metabolism by depleting gut bacterial populations that produce butyrate (i.e., *Clostridia* spp) [54]. Given the maintenance role of butyrate in colonic metabolism, we concluded that the switch of mitochondrial respiration in colonocytes to long-chain fatty acids might attribute to the reduction in colonic butyrate. Meanwhile, the researchers showed that physiological hypoxia could be re-established in the colonic surface of antibiotic-treated mice by feeding them a diet enriched in plant fiber or by activating the downstream pathway of butyrate (i.e., using a PPAR-γ agonist, such as 5-aminosalicylic acid) [48, 55, 56]. In line with the previous studies, supplementation of butyrate in drinking water significantly restored hypoxia in the colonic epithelium and reduced nitrate production, thereby decreasing the abundance of *Proteobacteria*, which contributed to the suppression of colonic IDO1 expression and peripheral Trp-Kyn metabolism (Fig. 6).

## Conclusions

In summary, our results highlighted the central role of the gut microbiota in HFD-induced dysregulation of Trp-Kyn metabolism. Mechanistic studies revealed that HFD-impaired mitochondrial bioenergetics caused metabolic reprogramming in colonocytes thriving the *Proteobacteria*, which stimulated colonic immune responses to upregulate the IDO1-mediated Kyn pathway. Butyrate supplementation promoted colonic mitochondrial functions to remodel the gut microbiota in mice with HFD, consequently ameliorating serum Kyn accumulation. These findings established the dominance of gut microbiota-colonocyte interactions in Trp metabolism and, therefore, provided new insights into therapies against diet-associated metabolic perturbations.

Methods.

### Animal experiments

All experiments in this study were approved by the Ethics Committee of the College of Veterinary Medicine, Northwest A&F University, and followed the Guide for the Care and Use of Laboratory Animals: Eighth Edition to carry out all experiments. The C57BL/6 J male mice were purchased from SPF Biotechnology Co., Ltd. (Beijing, China) at 4 weeks of age. Before the experiment, all mice were fed with the standard diet. During the experiment, animal caretakers and investigators were blinded to the group assignment of the mice.

To determine the impact of the high-fat diet on tryptophan (Trp) metabolism, the animals were randomly assigned to one of the two following diets: standard diet (Chow) (#XTHF0045-1-C) and high-fat diet (HFD) (#XTHF0045-1). The standard and high-fat feed were purchased from Jiangsu Xietong Medicine Bioengineering Co., Jiangsu, China, and kept at − 20 °C throughout the study. The Chow diet provided 10% calories from fat, 73% from carbohydrates, and 17% from protein. The HFD provided 63% of calories from fat, 20% from carbohydrates, and 17% from protein. After 4 weeks with the respective diets, the mice were humanely euthanized with 0.56% (v/v) pentobarbital sodium (10 mg/ml) and sacrificed by cervical dislocation, and biological samples were collected for the respective analysis.

Microbiota transplantation was performed to determine the causal role of the gut microbiota in HFD-mediated dysregulation of Trp metabolism. A high-dose antibiotic cocktail (Abx) was added in the drinking water for mice at the following concentration: neomycin (100 mg/l), streptomycin (50 mg/l), penicillin (100 mg/l), vancomycin (50 mg/l), and metronidazole (100 mg/l) [57]. Abx were freshly prepared on the day of treatment. Fecal microbiota transplantation (FMT) was performed according to the previous report [25]. Briefly, fecal pellets were collected from mice after 4 weeks with HFD and then pooled, mixed, and homogenized in PBS at 1 g feces/10 mL PBS. The mixture was centrifuged at 500 rpm for 5 min at 4℃, and the supernatant was collected and used for FMT. After persistent high-dose antibiotics treatment for 3 days, mice fed with standard diet received 150 µl of the supernatant by oral gavage once a day continuously for 28 days (C-FMT). HFD-fed mice were treated with the antibiotic cocktail to eliminate the innate gut microbiota (H-Abx). This antibiotic cocktail was shown to be sufficient to decrease mouse gut microbial load within hours and to target the full spectrum of bacteria, including gram-positive and gram-negative strains [57]. All antibiotics were purchased from Aladdin, China.

To verify the initiating role of *Proteobacteria* in the HFD-enhanced kynurenine (Kyn) metabolic pathway, the *E.coli* indicator strains were isolated from fresh feces of HFD mice. The fresh feces of HFD mice (0.2 g) were collected and immediately suspended in 1.8 mL PBS. The above mixture was centrifuged at 500 rpm for 5 min at 4℃ and plated on *E. coli* chromogenic medium (HB7001; Hopebio Co., Qingdao, China) at 36 °C for 24 h. *E. coli* colonies appear blue-green on the medium, while other bacteria are colorless or yellow. For further identification of bacteria, 16S rRNA sequencing and API kit assays were performed according to the method reported by Jang et al. [58]. Several *E. coli* clones were selected and cultured in Luria–Bertani (LB) broth at 36 °C with shaking. Cultures were centrifuged at 5000 × *g* for 20 min and washed twice with saline. Collected cells were suspended in saline, which was measured spectrophotometrically for optical density (OD) and diluted to OD_600_ = 0.8 (the cell concentration of the solution was approximated as 1 × 10^8^ CFU/ml). The diluted bacteria solution was transplanted by gavage into standard diet-fed mice (150 µl per mouse for sustained 3 days) (C-*E.coli*). Palmatine (P917112; Shanghai Macklin Biochemical Co., Ltd, Shanghai, China) was dissolved in saline and administered orally at the dose of 100 mg/kg for sustained 3 days prior to *E.coli* transplantation to inhibit IDO1 activity in the colon of mice (C-Pal).

Some mice with standard diet were treated with PPAR-γ antagonist GW9662 (5 mg/kg/day) for 7 days to verify the relationship between mitochondrial dysfunction and gut dysbiosis. Meanwhile, some HFD-fed mice were supplemented with sodium butyrate in drinking water (0.1 M) to investigate whether remodeling colonic mitochondrial bioenergetics could alleviate gut dysbiosis-induced dysregulation of Kyn metabolism. GW9662 and sodium butyrate were purchased from Aladdin, China.

### Metabolomics

The procedures of sample extraction and instrumental analysis were performed according to the previous protocol [59]. Notably, metabolomics was performed using Triple TOF 5600 + . The Waters Acquity UPLC HSS T3 column (100 Å, 1.8 μm, 2.1 mm × 100 mm) was used for the chromatographic separation.

All mass spectral data were analyzed based on R (version 4.1.3). MS1 *.wiff data were converted to *.mzXML files and MS2 *.wiff data were converted to *.mgf files in ProteoWizards MS Convert. The converted files were processed in R based on TidyMass (version 1.0.4) resulting in master peak tables aligning all samples [60]. Metabolites were annotated by the HMDB database [61]. The DESeq2 package (version 1.34.0) was used to identify the differential metabolites [62]. Chemical similarity enrichment analysis (ChemRICH) plots were generated to show which metabolites changed based on their chemical classes [63]. Quantitative metabolite set enrichment analysis (qMSEA) was performed using the R package MetaboAnalystR (version 3.3.0) to determine the biological function of altered metabolites [64].

### 16S rRNA amplicon and sequencing

Colonic contents were collected and snap-frozen in liquid nitrogen and transferred to storage at − 80C. V4 region of the 16S rRNA gene was amplified using primers 515F 5′-GTGCCAGCMGCCGCGGTAA-3′and 806R 5′-GGACTACHVGGGTWTCTAAT-3′ with barcodes. The sequencing steps were conducted by Magigene Technology Co., Ltd (Guangzhou, China). Sequencing data were processed following our previous protocols with modifications [40]. In detail, deblur method was adopted for sequence denoising, generating representative sequences and abundance table [65]. EZBioCloud-based Naive Bayes classifier was used to assign taxonomy to the representative sequences [66]. The diversity of gut microbiota was assessed by R package microeco (version 0.12.0) [67]. Linear discriminant analysis effect size (LEfSe) analysis was performed to identify differential bacterial taxa [68]. The correlation between bacterial taxa and diet-altered metabolites was calculated by LinkET in R (https://github.com/Hy4m/linkET). Random forest regression was conducted using the randomForest (version 4.7–1.1) and rfPermute (version 2.5.1) packages to generate variable importance plots to rank individual bacterial taxa [69].

### UID RNA sequencing of colonic tissue

Approximately 50 mg of colon tissue was used for RNA isolation. RNA extraction and sequencing process was performed by Seqhealth Technology Co., Ltd. (Wuhan, China). The de-duplicated consensus sequences were used for standard RNA-seq analysis. They were mapped to the reference genome of mice from ftp://ftp.ensembl.org/pub/release-87/fasta/mus_musculus/dna/ using STAR software with default parameters. Reads mapped to the exon regions of each gene were counted by featureCounts (Subread-1.5.1; Bioconductor) [70], and then RPKM was calculated. We detected differentially expressed genes between Chow and HFD groups with the DESeq2 (version 1.34.0) package [62], which was visualized by the EnhancedVolcano package in R (https://github.com/kevinblighe/EnhancedVolcano). Gene set enrichment analysis (GSEA) was performed on genes ranked by their differential expression using ClusterProfiler (version 4.2.2) [71] and GseaVis package in R (https://github.com/junjunlab/GseaVis). The abundance of the gene set was visualized by the ComplexHeatmap package (version 2.10.0) [72].

### Butyrate and lactate measurements

The measurements of butyrate and lactate in colonic contents were performed as previously described [73, 74].

### Nitrate measurements

Nitrate measurement in colonic tissue was performed by using a Total Nitrate Assay Kit (S0023, Beyotime Biotechnology, China), according to the manufacturer’s instructions.

### ATP measurements

ATP measurement in colonic tissue was performed by using an Enhanced ATP Assay Kit (S0027, Beyotime Biotechnology, China), according to the manufacturer’s instructions.

### Hypoxia staining

Hypoxia staining was performed as previously described [26]. Mice received a single intraperitoneal injection of 60 mg/kg pimonidazole HCl (HypoxyprobeTM-1 kit, Hypoxyprobe) 1 h before euthanasia. Colon samples were preserved in 4% paraformaldehyde in 0.1 M PB (pH = 7.40), and paraffin-embedded specimens were probed with mouse anti-pimonidazole monoclonal IgG1 followed by staining with Cy-3 conjugated goat anti-mouse antibody (ab97035, Abcam). DAPI was used for counterstaining. Samples were scored according to the degree of colonic epithelial hypoxia.

### Immunofluorescence assay

The primary antibody, indoleamine 2, 3 dioxygenase1 (IDO1), was purchased from Proteintech (66528–1-Ig). The immunofluorescence procedures were performed following our previous protocols [40].

### Quantification of *E.coli* abundance

The abundance of *E. coli* was determined using the Escherichia Coli Probe PCR Kit (Xin-Yu Biotechnology Co., Ltd, Shanghai, China) according to the manufacturer’s instructions.

### Statistical analysis

Student’s *t* test was used to analyze whether there were statistically significant differences between groups (*p*-value < 0.05). One-way analysis of variance (ANOVA) followed by Dunnett’s multiple comparison tests was used to determine whether there were significant differences between more than two treatments. The statistical significance of changes in hypoxia levels was determined using a non-parametric test (Wilcoxon test). A permutation test (999 times) was performed to assess the statistical significance of selected bacterial biomarkers. Statistical significance was indicated as follows: *, *p*-value < 0.05; **, *p*-value < 0.01; ***, *p*-value < 0.001. Data analysis was performed using R, and plots were generated using the Grafify package (version 2.3.0) in R. The sample size and type of statistical tests used are described in the legend of each figure.

## Supplementary Information

**Additional file 1:** **Figure S1. **The abundance of *Escherichia coli*. The abundance of *Escherichia coli *(*E.coli*) was determined by Escherichia Coli Probe PCR Kit. Data are represented as mean ± SD. *n* = 6 for each group. Statistical significance was assessed by independent samples *t*-test. NS not significant, * *p*-value ≤ 0.05, ** *p*-value ≤ 0.01, *** *p*-value ≤ 0.001. **Figure S2. **Correlation between gut bacterial taxa and serum kynurenine concentration. a The correlation between the abundance of gut bacterial taxa and serum kynurenine (Kyn) concentration was determined by the Pearson method. **Figure S3. **Antibiotic cocktail reduced the abundance of gut bacteria. a A high-dose antibiotic cocktail (Abx) was prepared to eliminate the gut bacteria. b Total bacterial amounts were quantified by *q*-PCR amplifying universal bacterial 16S rDNA genes (V3 region). c Oral gavage of Abx lasting three days eliminated more than 80% of the native gut microbiota (*n *= 9 for each group). Data are represented as mean ± SD. Statistical significance was assessed by independent samples *t*-test. NS not significant, * *p*-value ≤ 0.05, ** *p*-value ≤ 0.01, *** *p*-value ≤ 0.001. **Figure S4. **Principal component analysis score plot for assessing transcriptomes of colonic tissue between Chow and HFD mice. Permutational multivariate analysis of variance (PERMANOVA) by Adonis ( *p*-value = 0.016). PCA principal component analysis; Dim dimension. **Figure S5. **Transplantation of *E. coli *indicator strains. a *E. coli *indicator strains were isolated from the feces of HFD mice and were successfully transplanted into mice with a standard diet (*n *= 9 for each group). Data are represented as mean ± SD. Statistical significance was assessed by independent samples *t*-test. NS not significant, * *p*-value ≤ 0.05, ** *p*-value ≤ 0.01, *** *p*-value ≤ 0.001. **Figure S6. **HFD enhanced the oxidative stress in the colon. Persistent HFD enhanced β-oxidation of long- (a) and very-long-chain fatty acids (b) in colonic cells, which induced oxidative stress (c) to impair mitochondrial bioenergetics. NES normalized enrichment scores. **Figure S7. **HFD impaired the gut barrier. Sustained HFD inhibited the production of intestinal secretory mucin-2 (MUC2), an intestinal-type secretory mucin, in the colon. Nuclei were counterstained with DAPI (blue) (*n *= 9 slices from 3 mice). Data are represented as mean ± SD. Statistical significance was assessed by independent samples *t*-test. NS not significant, * *p*-value ≤ 0.05, ** *p*-value ≤ 0.01, *** *p*-value ≤ 0.001.

## Data Availability

The data included in this manuscript are available on reasonable request from the corresponding authors. Sequences generated as part of this study have been uploaded to the National Center for Biotechnology Information (NCBI), BioProject: PRJNA935508 and PRJNA935105. Metabolomics datasets reported in this paper have been deposited in the Open Archive for Miscellaneous Data (OMIX), China National Center for Bioinformation / Beijing Institute of Genomics, Chinese Academy of Sciences (accession: OMIX003531). The computer code used in this study is available at https://gitee.com/neokie/r-code-for-microbiome-analysis/tree/master.

## Abbreviations

Trp: Tryptophan
Kyn: Kynurenine
HFD: High-fat diet
LPS: Lipopolysaccharide
IDO1: Indoleamine 2,3-dioxygenase 1
*E.coli*: *Escherichia coli*
3-HK: 3-Hydroxykynurenine
FMT: Fecal microbiota transplantation
PCA: Principal component analysis
PERMANOVA: Permutational multivariate analysis of variance
ChemRICH: Chemical similarity enrichment analysis
qMSEA: Quantitative metabolite sets enrichment analysis
rRNA: Ribosomal RNA
LDA: Linear discriminant analysis
LEfSe: Linear discriminant analysis effect size
Abx: Antibiotic cocktail
GSEA: Gene set enrichment analysis
PPAR-γ: Peroxisome proliferator-activated receptor-γ
SCFAs: Short-chain fatty acids
ATP: Adenosine triphosphate
ROS: Reactive oxygen species
MUC2: Mucin-2, TLRs: Toll-like receptors
