# Supplementary material for: High-fat diet-disturbed gut microbiota-colonocyte interactions contribute to dysregulating peripheral tryptophan-kynurenine metabolism
Source: Microbiome. 2023 Jul 19;11:154. doi: 10.1186/s40168-023-01606-x (PMC10355067; doi:10.1186/s40168-023-01606-x)
Supplement: Supplementary file 2 — Additional file 1: Figure S1. The abundance of Escherichia coli. The abundance of Escherichia coli (E.coli) was determined by Escherichia Coli Probe PCR Kit. Data are represented as mean ± SD. n = 6 for each group. Statistical significance was assessed by independent samples t-test. NS not significant, * p-value ≤ 0.05, ** p-value ≤ 0.01, *** p-value ≤ 0.001. Figure S2. Correlation between gut bacterial taxa and serum kynurenine concentration. a The correlation between the abundance of gut bacterial taxa and serum kynurenine (Kyn) concentration was determined by the Pearson method. Figure S3. Antibiotic cocktail reduced the abundance of gut bacteria. a A high-dose antibiotic cocktail (Abx) was prepared to eliminate the gut bacteria. b Total bacterial amounts were quantified by q-PCR amplifying universal bacterial 16S rDNA genes (V3 region). c Oral gavage of Abx lasting three days eliminated more than 80% of the native gut microbiota (n = 9 for each group). Data are represented as mean ± SD. Statistical significance was assessed by independent samples t-test. NS not significant, * p-value ≤ 0.05, ** p-value ≤ 0.01, *** p-value ≤ 0.001. Figure S4. Principal component analysis score plot for assessing transcriptomes of colonic tissue between Chow and HFD mice. Permutational multivariate analysis of variance (PERMANOVA) by Adonis ( p-value = 0.016). PCA principal component analysis; Dim dimension. Figure S5. Transplantation of E. coli indicator strains. a E. coli indicator strains were isolated from the feces of HFD mice and were successfully transplanted into mice with a standard diet (n = 9 for each group). Data are represented as mean ± SD. Statistical significance was assessed by independent samples t-test. NS not significant, * p-value ≤ 0.05, ** p-value ≤ 0.01, *** p-value ≤ 0.001. Figure S6. HFD enhanced the oxidative stress in the colon. Persistent HFD enhanced β-oxidation of long- (a) and very-long-chain fatty acids (b) in colonic cells, which induced oxidative [file 40168_2023_1606_MOESM1_ESM.pdf]

High-fat diet-disturbed gut microbiota-colonocyte interactions contribute to dysregulating peripheral tryptophan-kynurenine metabolism

Penghao Sun[1#], Mengli Wang[1#], Yong-Xin Liu[2], Luqi Li[3], Xuejun Chai[4*], Wei Zheng[5], Shulin Chen[1], Xiaoyan Zhu[1*], Shanting Zhao[1*]

# These authors contributed equally to this work.

Affiliations

1. College of Veterinary Medicine, Northwest A&F University, Yangling, China
2. Shenzhen Branch, Guangdong Laboratory of Lingnan Modern Agriculture, Genome Analysis Laboratory of the Ministry of Agriculture and Rural Affairs, Agricultural Genomics Institute at Shenzhen, Chinese Academy of Agricultural Sciences, Shenzhen, Guangdong 518120, China
3. Life Science Research Core Services, Northwest A&F University, Yangling, China
4. College of Basic Medicine, Xi'an Medical University, Xi'an, China
5. College of Resources and Environment Sciences, Northwest A&F University, Yangling, China

Corresponding authors

Shanting Zhao, Tel: 18700975432, E-mail: zhaoshanting@nwsuaf.edu.cn

Xuejun Chai, Tel: 13759959851, E-mail: xchai@xiyi.edu.cn

Xiaoyan Zhu, Tel: 18302907099, E-mail: xyzhu0922@nwsuaf.edu.cn

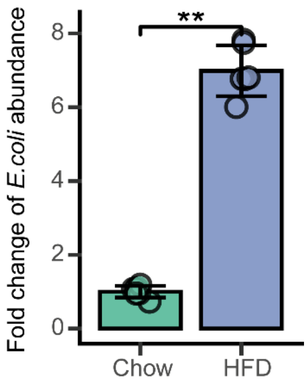

Figure S1. The abundance of *Escherichia coli*. The abundance of *Escherichia coli* (*E.coli*) was determined by Escherichia Coli Probe PCR Kit. Data are represented as mean ± SD. n = 6 for each group. Statistical significance was assessed by independent samples *t*-test. NS not significant, * *p*-value ≤ 0.05, ** *p*-value ≤ 0.01, *** *p*-value ≤ 0.001.

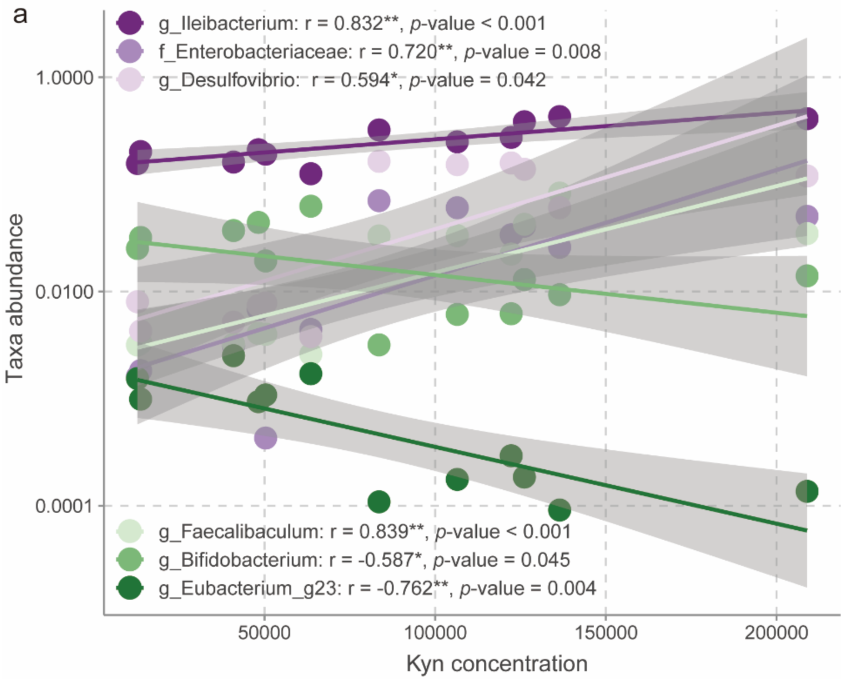

Figure S2. Correlation between gut bacterial taxa and serum kynurenine concentration. a The correlation between the abundance of gut bacterial taxa and serum kynurenine (Kyn) concentration was determined by the Pearson method.

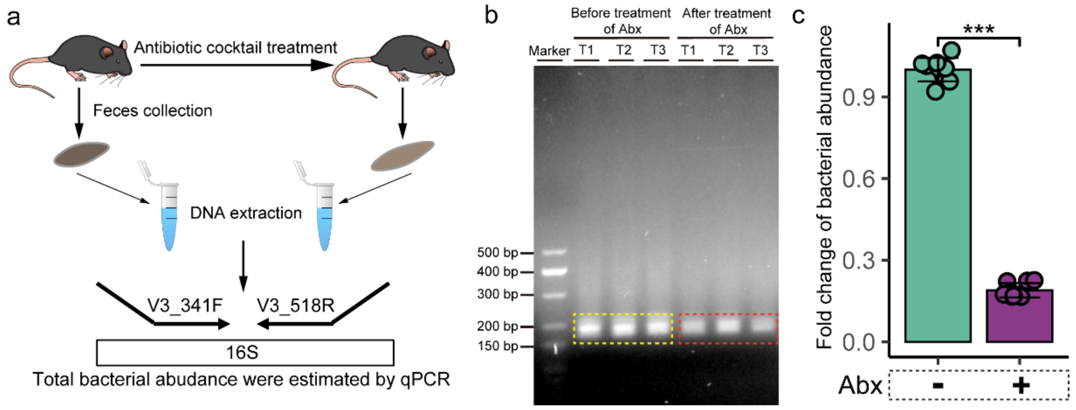

Figure S3. Antibiotic cocktail reduced the abundance of gut bacteria. a A high-dose antibiotic cocktail (Abx) was prepared to eliminate the gut bacteria. **b** Total bacterial amounts were quantified by *q*-PCR amplifying universal bacterial 16S rDNA genes (V3 region). **c** Oral gavage of Abx lasting three days eliminated more than 80% of the native gut microbiota ($n = 9$ for each group). Data are represented as mean ± SD. Statistical significance was assessed by independent samples *t*-test. NS not significant, * p-value ≤ 0.05, ** p-value ≤ 0.01, *** p-value ≤ 0.001.

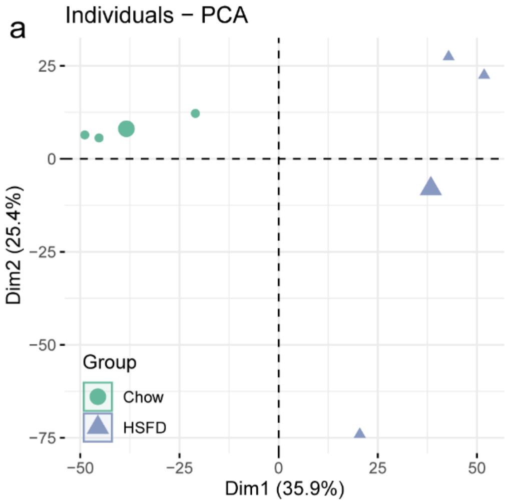

Figure S4. Principal component analysis score plot for assessing transcriptomes of colonic tissue between Chow and HFD mice. Permutational multivariate analysis of variance (PERMANOVA) by Adonis (p-value = 0.016). PCA principal component analysis; Dim dimension.

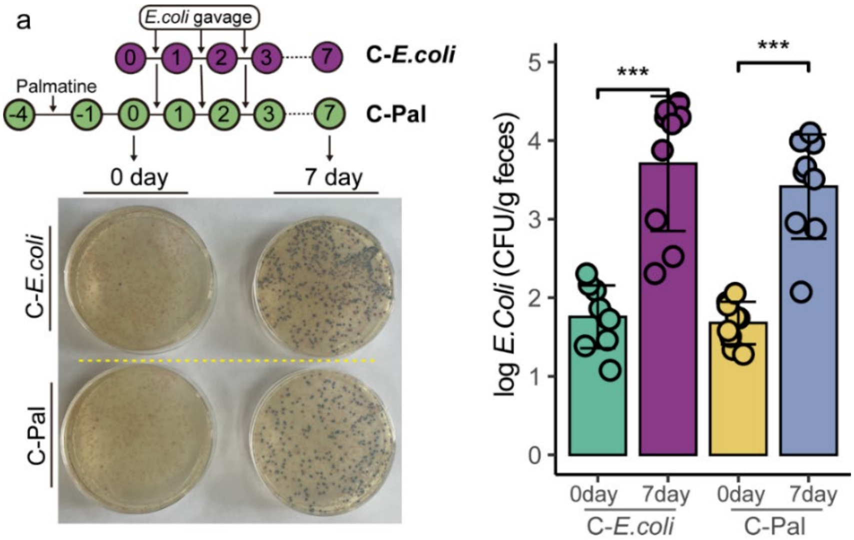

Figure S5. Transplantation of *E. coli* indicator strains. a *E. coli* indicator strains were isolated from the feces of HFD mice and were successfully transplanted into mice with a standard diet (n = 9 for each group). Data are represented as mean ± SD. Statistical significance was assessed by independent samples t-test. NS not significant, * p-value ≤ 0.05, ** p-value ≤ 0.01, *** p-value ≤ 0.001.

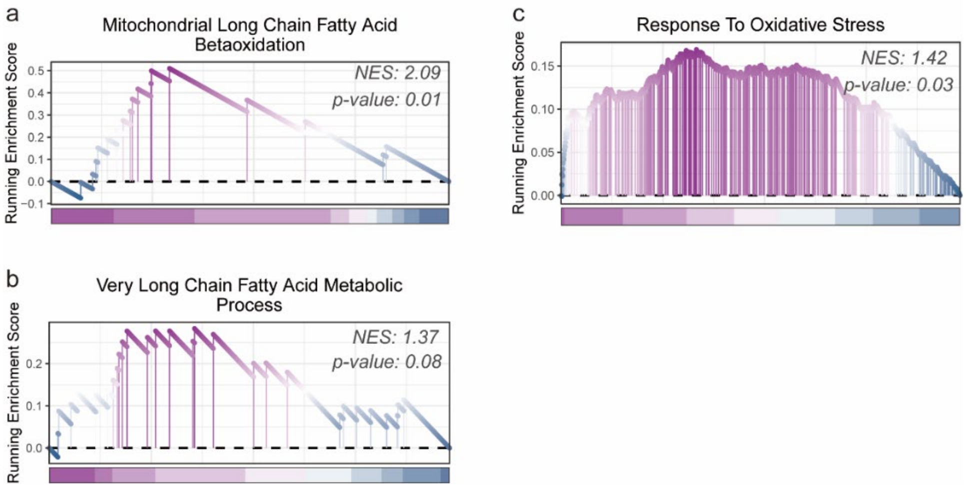

Figure S6. HFD enhanced the oxidative stress in the colon. Persistent HFD enhanced β-oxidation of long- (**a**) and very-long-chain fatty acids (**b**) in colonic cells, which induced oxidative stress (**c**) to impair mitochondrial bioenergetics. NES normalized enrichment scores.

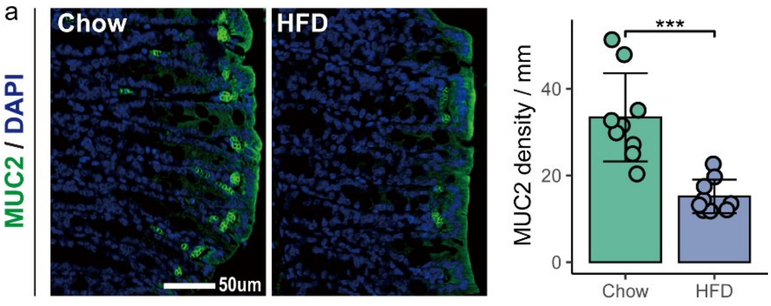

Figure S7. HFD impaired the gut barrier. Sustained HFD inhibited the production of intestinal secretory mucin-2 (MUC2), an intestinal-type secretory mucin, in the colon. Nuclei were counterstained

with DAPI (blue) ($n = 9$ slices from 3 mice). Data are represented as mean ± SD. Statistical significance was assessed by independent samples t-test. NS not significant, * p-value ≤ 0.05, ** p-value ≤ 0.01, *** p-value ≤ 0.001.
